# The effect of nucleus basalis magnocellularis deep brain stimulation on memory function in a rat model of dementia

**DOI:** 10.1186/s12883-016-0529-z

**Published:** 2016-01-12

**Authors:** Ji Eun Lee, Da Un Jeong, Jihyeon Lee, Won Seok Chang, Jin Woo Chang

**Affiliations:** Brain Korea 21 Plus Project for Medical Science & Brain Research Institute, Yonsei University College of Medicine, Seoul, Korea; Department of Neurosurgery, Severance Hospital, Yonsei University College of Medicine, Seoul, 120-752 Republic of Korea

**Keywords:** Nucleus basalis magnocellularis, Deep brain stimulation, Spatial memory, Medial prefrontal cortex, Hippocampus

## Abstract

**Background:**

Deep brain stimulation has recently been considered a potential therapy in improving memory function. It has been shown that a change of neurotransmitters has an effect on memory function. However, much about the exact underlying neural mechanism is not yet completely understood.

We therefore examined changes in neurotransmitter systems and spatial memory caused by stimulation of nucleus basalis magnocellularis in a rat model of dementia.

**Methods:**

We divided rats into four groups: Normal, Lesion, Implantation, and Stimulation. We used 192 IgG-saporin for degeneration of basal forebrain cholinergic neuron related with learning and memory and it was injected into all rats except for the normal group. An electrode was ipsilaterally inserted in the nucleus basalis magnocellularis of all rats of the implantation and stimulation group, and the stimulation group received the electrical stimulation. Features were verified by the Morris water maze, immunochemistry and western blotting.

**Results:**

All groups showed similar performances during Morris water maze training. During the probe trial, performance of the lesion and implantation group decreased. However, the stimulation group showed an equivalent performance to the normal group. In the lesion and implantation group, expression of glutamate acid decarboxylase65&67 decreased in the medial prefrontal cortex and expression of glutamate transporters increased in the medial prefrontal cortex and hippocampus. However, expression of the stimulation group showed similar levels as the normal group.

**Conclusion:**

The results suggest that nucleus basalis magnocellularis stimulation enhances consolidation and retrieval of visuospatial memory related to changes of glutamate acid decarboxylase65&67 and glutamate transporter.

## Background

Alzheimer’s disease (AD) is a progressive and irreversible neurodegenerative disease accompanied by decline of memory and cognitive function [1]. Degeneration of cholinergic basal forebrain neurons is one of the common features of AD [2]. It has been reported that degeneration of basal forebrain cholinergic neurons (BFCN) and the decrease of cholinergic projections could be an important factor characterizing the cognitive decline and functional impairment that characterizes this disorder [2–4].

As an effective surgical treatment, deep brain stimulation (DBS) has demonstrated its efficacy for the treatment of a variety of movement disorders [5]. Recently, there is growing evidence from laboratory and clinical trials that electrical stimulation at memory associated structures enhances cognitive functions, and DBS has increasingly been considered as a potential therapy because of its recent effects in improving memory function [6, 7]. However, there is currently limited evidence at best that DBS improves memory functioning in humans. Stimulation of the entorhinal region in patients with dementia could improve patient’s spatial memory performance [8]. In addition, electrical stimulation of the fornix affects hippocampus-dependent memory [9]. Although DBS is being evaluated as a promising therapy for diseases related to memory impairment, much about the underlying exact neural mechanism of DBS is not yet completely understood. Therefore, studies are needed to characterize not only the effective regions and stimulation parameters for DBS causing memory improvement, but also the mechanisms connecting memory enhancement and changes of the neural circuit caused by stimulation.

In this study, we used 192 IgG-saporin for degeneration of BFCN to make a memory impaired rat model mimicking cholinergic denervation of AD. Composed of a monoclonal antibody, 192 IgG-sapoin, has a low affinity for the rat nerve growth factor receptor p75 located on cholinergic cell bodies of the basal forebrain. And it contains a ribosomal inactivating protein called saporin [10–12]. Therefore, 192 IgG-saporin injection to the intraventricular cause selective degeneration of BFCN closely related with spatial learning and memory [13, 14].

Nucleus basalis magnocellularis (NBM) in the basal forebrain has mostly cholinergic neurons, and also it has a few of noncholinergic neurons such as GABAergic and glutamatergic [15]. It has mainly projections to the neocortex, amygdala, and thalamus [16, 17]. Substantial evidence suggests that the NBM plays an important role in neural activities such as learning and memory [11, 16, 18]. NBM lesions by ibotanic acid resulted in deficits in the acquisition of spatial memory tasks, and produced a profound selective disturbance in recent memory [19, 20]. Meanwhile, a research on a two-way active avoidance task (a test of associative memory) suggests the stimulation of NBM can improve the acquisition memory [21, 22]. Especially, the high frequency stimulation by regulating neurotransmitters improves memory function. A study suggests that high frequency stimulation in the dorsal striatum is effects on response learning and on GABA levels [23]. And also, NBM stimulation in patients with Parkinson-dementia syndrome caused improvement of cognitive function [7]. In the present study, we examined whether unilateral electrical stimulation of the NBM improves memory in a rat model that mimicking the basal forebrain cholinergic deficits of AD.

## Methods

### Animals

All experiments were conducted according to the guidelines of the Institutional Animal Care and Use Committee of Yonsei University and Institute of Laboratory Anima Resources commission on Life Sciences National Research Council, USA. This study received approval of Institutional Animal Care and Use Committee (IACUC number: 2014-0206). Every effort was made to minimize animal suffering and to reduce the number of rats used. Twenty-five male Sprague–Dawley rats were used that weighed 190 − 210 g (6 week old) at the beginning of the studies. They were housed in clear plastic cages in a temperature- and humidity-controlled room with a 12-h light/12-h dark cycle. Rats were randomly assigned to one of four experimental groups before surgery. The normal group (*n* = 6) underwent no surgical procedures. The lesion group (*n* = 7) had intraventricular administration of 192 IgG-saporin. The implantation group (*n* = 7) and stimulation group (*n* = 5) had intraventricular administration of 192 IgG-saporin and implantation of an electrode in the NBM. The stimulation group had unilateral electrical stimulation.

### Administration of 192 IgG-saporin

Rats in the three experimental groups, but not the normal group, were anesthetized with a mixture of ketamine (75 mg/kg), acepromazine (0.75 mg/kg), and xylazine (4 mg/kg), and secured in a stereotaxic frame. Eight microliters (0.63 μg/μl) of 192 IgG-saporin (Chemicon, Temecula, CA, USA) were bilaterally injected into the lateral ventricle following coordinates relating to Bregma (AP - 0.8 mm, ML ± 1.2 mm, DV - 3.4 mm). The solutions were delivered at a rate of 1 μl/min and diffused for 5 min after each injection.

### Electrode implantation

Right after the administration of 192 IgG-saporin, an electrode (SNEX-100, contact diameter 0.1 mm, shaft diameter 0.25 mm, impedance 0.7–1.5 MΩ; David Kopf Instruments, Tujunga, CA, USA) was inserted into the right NBM following coordinates relating to Bregma (AP -1.32 mm, ML + 2.8 mm, DV -7.4 mm) of the rats in the implantation and stimulation group. Dental cement (Long Dental Manufacturing, Wheeling, IL, USA) was used to firmly fix the electrode in place to the skull surface.

### Electrical stimulation

The stimuli were bipolar and electrical stimuli (120 Hz, 90 μs, 1 V) delivered via a Grass A-300 pulsemaster (Grass Instrument, Quincy, MA, USA). Stimulation parameters were monitored in real time at the beginning and end of stimulation using an oscilloscope (HDS 1022 M, Owon, Korea). Rats were unilaterally stimulated daily beginning one week after surgery until the start of behavioral testing (1 h per day, 1 week in total).

### Morris water maze (MWM)

The MWM test began 2 weeks after surgery. The test consisted of 5 consecutive days of training trials and 1 day of a probe trial. A circular dark water maze tank (2 m diameter, 50 cm deep) located in a dimly lit room, was filled to a depth of 40 cm with water (maintained at 25 °C). A circular escape platform (15 cm diameter) was submerged 2 cm below the water surface. In the training trials, rats were given four acquisition trials per day with four different starting positions that were equally distributed around the perimeter of the maze. The test required the rats to swim to the hidden platform guided by distal spatial cues. Throughout acquisition, the platform was located in a fixed position in the center of the southwest quadrant of the pool. For each trial, rats were released randomly from each of the four cardinal points from the edge of the pool and gently released facing the wall. Rats were given a maximum of 60 s to find the platform. After finding the platform, rats were allowed to remain on the platform for 10 s and placed in their home cages. Animals failing to find the platform in 60 s were guided by an experimenter, then placed on the platform, and allowed to rest for 10 s. After completion of training, rats were returned to their home cages for 2 days until the probe trial. The probe trial consisted of a 60 s free swim period without a platform, during which the time spent in the target quadrant and the number of platform crossings was recorded.

### Choline acetyltransferase (ChAT) immunohistochemistry

After behavioral testing, three out of six rats from the normal group, three out of the seven rats from the lesion group, three out of seven rats from the implantation group, two out of five rats from the simulation group were perfused with cold 4 % paraformaldehyde in phosphate buffer saline (pH 7.2). Their brains were removed, post-fixed, and transferred to 30 % sucrose. The brains were cut into 30 μm coronal sections using a freezing microtome. The cryoprotection solution consisted of 0.1 M phosphate buffer (pH 7.2), 30 % sucrose, 1 % polyvinylpyrrolidone, and 30 % ethylene glycol. Tissue was stained with cresyl violet to confirm correct placement of the needle tracks. To detect cholinergic cells, tissue was immunohistochemically processed using polyclonal antibodies against choline acetyltransferase (ChAT; 1:100; cat# AB144P, Chemicon). The sections were stained using the avidin-biotin complex method (Vector Labs, Burlingame, CA, USA) with diaminobenzidinetetrahydrochloride as the substrate. Also fluorescence immunohistochemistry was performed with ChAT primary antibody (1:50; cat# AB144P, Chemicon) and Texas Red (1:400; cat# ab6883, abcam). Anatomical landmarks from a stereotaxic atlas [24] were used to localize the medial septum (MS).

### Acetylcholinesterase (AChE) assay

Three out of six rats from the normal group, four out of the seven rats from the lesion group, four out of seven rats from the implantation group, three out of five rats from the simulation group were decapitated with a guillotine, and their brains were quickly removed to acquire protein for AChE assay and Western blot. The medial prefrontal cortex and hippocampus were dissected with fine forceps from 1 mm thick coronal brain slices. The samples were homogenized in lysis buffer (Intron, Seongnam, Korea) and centrifuged for 10 min at 12,000 rpm, and the protein in the supernatant was measured using the bicinchoninic acid protein assay reagent kit (Pierce, Rockford, IL, USA). The enzymatic activity of AChE was determined using the method of Ellman et al. [25] with some modifications. Twenty microliter triplicate samples identical to those used in western blot analyses were mixed with a reaction mixture [0.2 mM dithiobisnitrobenzoic acid (Sigma-Aldrich, St. Louis, MO, USA), 0.56 mM acetylthiocholine iodide (Sigma-Aldrich), 10 μM tetraisopropylpyrophosphoramide (Sigma-Aldrich), 39 mM phosphate buffer; pH 7.2] at 37 °C. After 30 min, the optical density was measured at 405 nm.

### Western blot

To acquire western blot data, proteins were separated using a 10 − 15 % sodium dodecyl sulfate-polyacrylamide gel and transferred onto polyvinylidene fluoride membranes. The membranes were incubated with blocking buffer [5 % nonfat dry milk in phosphate-buffered saline containing 0.05 % Tween-20 (PBST)] for 1 h at room temperature. They were then incubated with the indicated antibodies overnight at 4 °C and washed three times with PBST. The membranes were incubated with corresponding secondary antibodies for 1 h at room temperature. After washing with PBST, proteins were detected with enhanced chemiluminescence solution (Pierce) and LAS-4000 (Fujifilm, Tokyo, Japan). The intensity of each band was determined using an analysis system (Multi Gauge version 3.0, Fujifilm, Tokyo, Japan). The membranes were incubated with antibodies to glutamate acid decarboxylase 65/67 (GAD 65&67) (1:500; Millipore, Temecula, CA, USA), glutamate transporter (GT) (1:4000; Abcam, Cambridge, UK), and β-actin (1:1000; Sigma-Aldrich).

### Statistical analysis

The results of all experiments were expressed as a percentage of the values for the normal group. The results of the western blots were normalized to β-actin for each sample and expressed as a percentage of the normal values. One-way ANOVA was used for overall analysis of experiments. ANOVA followed by least significant difference was used as a post hoc test at each time point for statistical analysis. The p values less than 0.05 were considered statistically significant. All statistical analyses were performed by PASW (version 18; SPSS Inc., Chicago, IL, USA).

## Results

### The location of MS and degeneration of ChAT-immunopositive neurons by 192 IgG-saporin

**Fig. 1:**
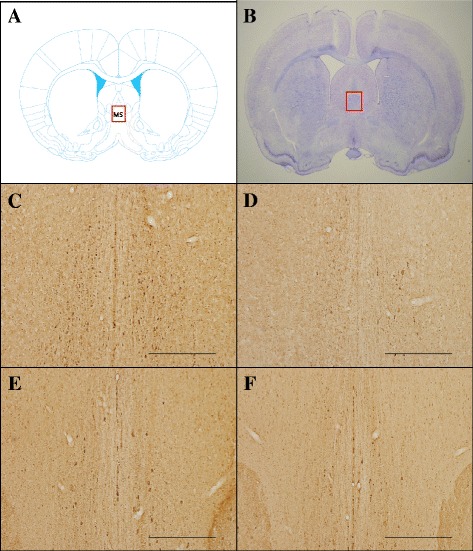
Representative photographs are showing the location of MS and the effect of cholinergic lesions on the MS. Figure (**a**) shows the location of MS in Atlas [24] and Figure (**b**) shows the location of MS in a brain slice stained with cresyl violet. The normal group had numerous ChAT-immunopositive neurons (**c**). The lesion (**d**), implantation (**e**), and stimulation (**f**) groups show a loss of cholinergic neurons. Scale bar represents 500 μm

**Fig. 2:**
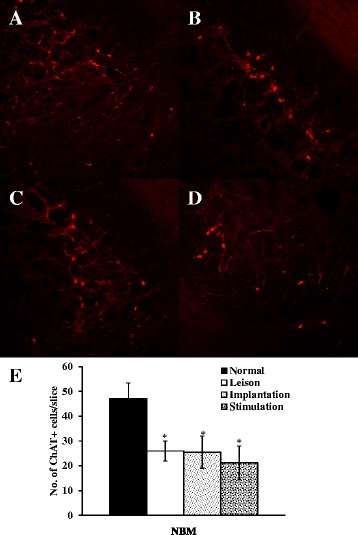
Representative photographs showing ChAT-immunopositive neurons in the NBM. Numerous ChAT-immunopositive neurons (*red*) were showing in the normal group (**a**). However, cholinergic neurons were significantly damaged by 192 IgG-saporin (lesion group, **b**; implantation group, **c**; stimulation group, **d**) The number of ChAT-immunopositive cells were significantly decreased  in lesion, implantation and stimulation group compared with normal group (e).

Figure 1a and b shows the location of MS in the atlas [24] and in a brain slice stained with cresyl violet. The 192 IgG-saporin injections produced denervation of ChAT-immunopositive neurons in the MS (Fig. 1). In normal rats (C), the ChAT-immunopositive neurons were evenly distributed in the MS. By contrast, lesion (D), implantation (E), and stimulation (F) groups showed a remarkable decrease. The number of ChAT-immunopositive cells of the normal group was 125 ± 10.11. However, it was significantly decreased by administration of 192 IgG-saporin (lesion group, 18.3 ± 3.3; implantation group, 25 ± 4.5; stimulation group, 28 ± 5.6). In addition, 192 IgG-saporin damaged ChAT-immunopositive cells in the NBM (Fig. 2). The normal group (A) averagely had 47 ± 6.2 ChAT positive cells whereas 192 IgG-saporin injection groups (lesion, B; implantation, C; stimulation, D) remarkably decreased ChAT positive cells (lesion group, 26 ± 4.0; implantation group, 25.5 ± 6.4; stimulation group, 21.1 ± 6.8).

### Confirmation of the location of the electrode in the NBM

**Fig. 3:**
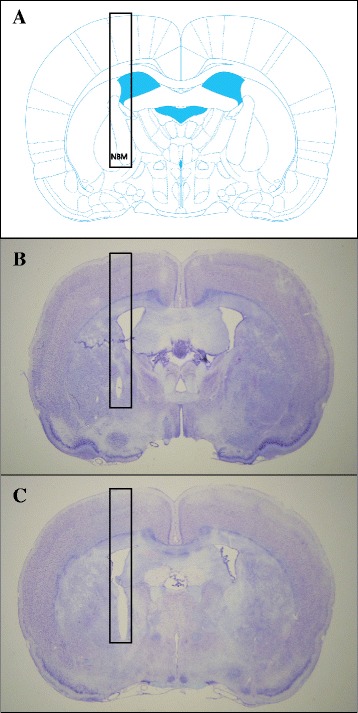
The location of the NBM in the rat brain atlas (**a**) and in a brain slice stained with cresyl violet in implantation (**b**) and stimulation group (**c**). The rectangles black box designates the insertion track of the electrode

We confirmed the location of the inserted electrode in the NBM using cresyl violet staining. Figure 3a shows the location of NBM in a atlas. And also, Fig. 3b and c shows the location of NBM in the implantation and stimulation group, respectively. Although the electrodes were very close to the pallidum and internal capsule, there were no side effects such as seizure, abnormal behavior when we implant the electrodes and stimulate the nucleus basalis.

### The performances of all groups in the MWM

**Fig. 4:**
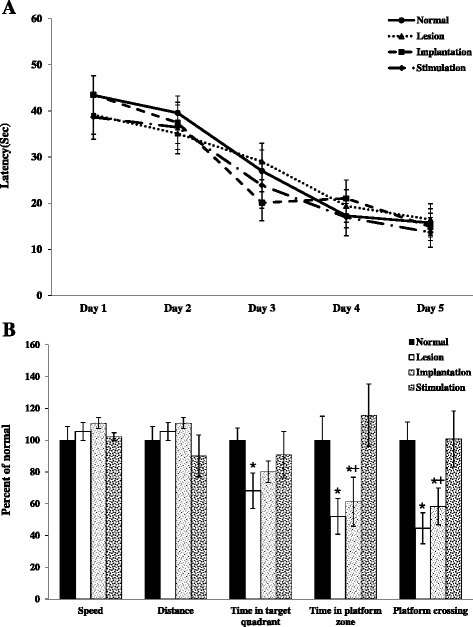
Spatial memory performance during training trials (**a**) and the probe trial (**b**) in the MWM. The 5 days of training trial, the latency to find platform gradually decreased (**a**). And the percentage of stimulation group to find platform is significantly higher than lesion and implantation group (**b**)

On the last day of training trials, all groups found the platform in 20 s, which was evidence of learning the platform location (Fig. 4a). During the probe trial, all groups showed similar motor-related behaviors, evidenced by equivalent swim distances and speeds. These findings suggest no effect of cholinergic lesion, electrode implantation, or electrical stimulation on motor function (Fig. 4b). Compared to the normal group, the lesion (no implantation) group showed less time in the target quadrant, less time in the platform zone, and fewer platform crossings (*p < 0.05). The stimulation group showed similar performance as compared with the normal group in terms of time spent in the target quadrant and number of platform crossings. Notably, there were statistically significant differences between the implantation and stimulation group in time on the platform zones and platform crossings (+*p* < 0.05).

### AChE activity in the medial prefrontal cortex (mPFC) and the hippocampus

**Fig. 5:**
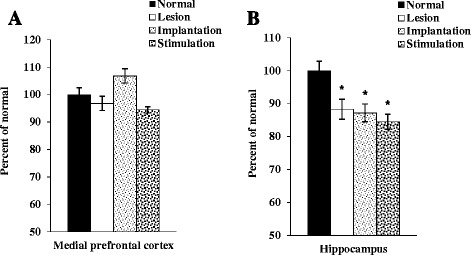
AChE activity in the mPFC (**a**) and hippocampus (**b**). Indices are expressed as the percentage of normal group values (mean ± standard error of the mean)

There were no significant differences of AChE activity in the mPFC among groups (Fig. 5a). In addition, there were no differences among groups in AChE activity in the hippocampus, except for the normal group (Fig. 5b).

### The expressions of GAD 65&67 and GT in the mPFC and hippocampus

**Fig. 6:**
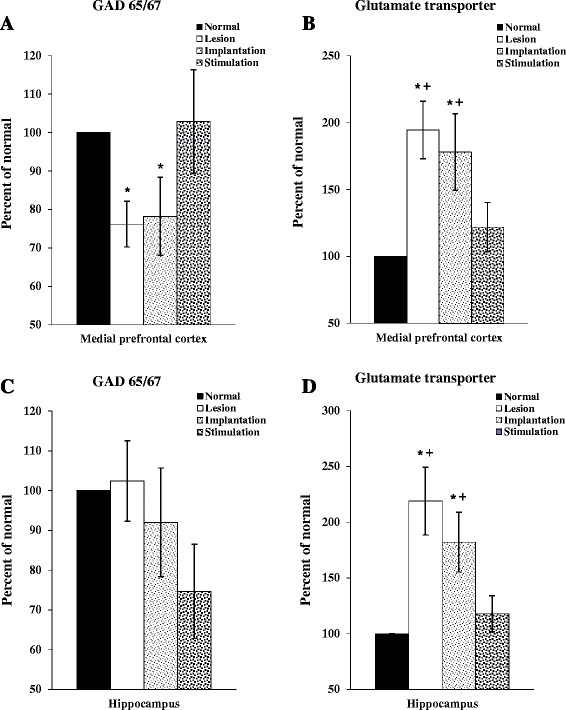
The expression of GAD65&67 and GT in the mPFC and hippocampus. The expression of GAD65&67 (**a**) and GT (**b**) in the mPFC. The expression of GAD65&67 (**c**) and GT (**d**) in the hippocampus (**d**). Unlike the lesion and implantation group, the stimulation group showed a similar percentage as compared to the normal group except for the expression of GAD65&67 in the hippocampus. The results of the western blots analyses were normalized to β-actin for each sample and expressed as a percentage of normal group values

In the mPFC, expression of GAD 65&67 in the lesion and lesion-implantation groups was decreased compared with the normal group. However, the stimulation group showed a similar level as the normal group (Fig. 6a). In the hippocampus, the expression level of GAD 65/67 was decreased to 25.32 % in animals receiving DBS. However, there were no significant differences of GAD65&67 expressions among groups in the hippocampus (Fig. 6c). The lesion and implantation groups showed a significant increase in expression of GT both in the mPFC and hippocampus (Fig. 6b and d) (*p < 0.05). However, the stimulation group showed a similar GT expression as compared to the normal group in the both regions. And also, the stimulation group showed notably decreased expression of GT both mPFC and hippocampus as compared with the implantation group (Fig. 6b and d) (+*p* < 0.05).

## Discussion

The results of the present study showed improved spatial memory performance in the MWM after stimulation of the NBM, suggesting that stimulation of the NBM affects enhancement of visuospatial memory. Our results support a recent clinical study which reported slight improvement or stabilization of AD-associated symptoms by NBM-DBS [26]. In addition, we examined changes of GAD 65&67 and GT expression after stimulation of the NBM, which showed that NBM regulates changes of the GABA (Gamma-Amino Butyric Acid) and glutamate systems.

The interactions between the mPFC and hippocampus are essential for learning and memory of a visuospatial memory test such as the MWM [27]. A previous study reported that functional disconnection between the mPFC and hippocampus impaired spatial working memory in rats [28]. Therefore, interaction between the two structures is necessary [29]. The mPFC is essential for consolidation and retrieval, and the hippocampus is crucial for the acquisition in MWM [27, 30–33]. In this study, there were differences among groups in the probe trial in the MWM but not in the training trial. Taken together, the results show that mPFC is more affected than the hippocampus by stimulation of the NBM.

GABA is closely involved in memory function [34, 35]. Injections of the GABA blocker picrotoxin may interfere with consolidation [36], and an increase of GABA by chemotherapy might enhance the learning ability of retarded or emotionally disturbed children [37]. Reduced GABA activity by cholinergic lesion was increased by NBM stimulation to a similar level as the normal group in the mPFC in this study. Previous studies suggested that GABA is more closely involved in memory consolidation and retrieval phases than the memory acquisition phase, and suggested that GABA is engaged in earlier period of consolidation, whereas acetylcholine (Ach) affects relatively remote period of memory consolidation in septo-hippocampal [38]. The results of this study showed a memory deficit by decreasing GAD level after lesion by 192-IgG saporin, and memory enhancement by increasing GAD level after NBM stimulation. The GAD level changed by NBM stimulation seems to cause improvement of recent retention memory in the mPFC that is known to be involved consolidation and retrieval during probe test of MWM test. Actually, some studies suggest that NBM stimulation may also activate neurotransmitter systems such as γ-aminobutyric acid neurons that send projections to the cortex [39, 40].

It is known that denervation of BFCN by 192 IgG-saporin increases glutamatergic response in the hippocampus [41]. Glutamate transporters of glial cell allow neighboring synapses to operate more independently, and control the postsynaptic response to high frequency bursts of action potentials [42]. In this study, NBM stimulation reduced GT overexpression by lesions to a similar level as the normal group, which seemed to have an effect on the spatial memory. However, there should be further studies to understand the exact mechanisms of this process.

Ach is regarded as an essential factor for memory processing and cognitive functioning [43, 44]. We showed spatial memory improvement, although there were no changes of AchE activity in the mPFC and hippocampus after stimulation. Ach is mainly associated with the memory that is involved relatively remote period. In a study, the cholinergic lesioned group maintained their consolidation memory in until after 5 day, while the GABAergic lesioned group did not maintained their memory until 5 day in MWM [38]. The MWM in this study includes relatively recent retention periods. Therefore, the spatial memory improvement in the present study may have been affected by other factors rather than Ach. However, unless we directly confirm the change in the amount of acetylcholine, cholinergic effects seems not be excluded. In addition, the effects of cholinergic neurons which remained in the stimulation site could not be excluded.

## Conclusions

NBM stimulation enhances consolidation and retrieval of visuospatial memory related to changes of GAD and GT level in the mPFC. However, it is unclear how electrical stimulation affects changes of the neurotransmitter system, and how the changes are involved in visuospatial memory. Therefore, future studies need to examine the exact mechanisms connecting electrical stimulation and the various neurotransmitter systems.

## Abbreviations

DBS: deep brain stimulation
AD: Alzheimer’s disease
BFCN: basal forebrain cholinergic neurons
NBM: nucleus basalis magnocellularis
MWM: Morris water maze
ChAT: choline acetyltransferase
AchE: acetylcholinesterase
MS: medial septum
PBST: phosphate buffered saline with Tween20
GAD: glutamate acid decarboxylase
GT: glutamate transporter
Ach: acetylcholine
mPFC: medial prefrontal cortex
